# Digital literacy competence among dental students: an assessment using a DigComp 2.2-based instrument

**DOI:** 10.1186/s12909-026-08916-2

**Published:** 2026-05-25

**Authors:** Mohammad Shooriabi, Farideh KaabOmeir, Sediqeh Modarres Mousavi, Mehrdad Amjadi

**Affiliations:** 1 Department of Oral Medicine, School of Dentistry, Ahvaz Jundishapur University of Medical Sciences, Ahvaz, Iran; 2 Department of Medical Library & Information Science, School of Allied Medical Science, Ahvaz Jundishapur University of Medical Sciences, Ahvaz, Iran; 3 Department of Oral Medicine, Faculty of Dentistry, Ahvaz Jundishapur University of Medical Sciences, Ahvaz, Iran; 4 School of Dentistry, Ahvaz Jundishapur University of Medical Sciences, Ahvaz, Iran

**Keywords:** Information Literacy, Computer Literacy, Dental Students, Self-Assessment, Educational Measurement

## Abstract

**Background:**

In the digital age, university students are expected to possess adequate technological competencies to successfully navigate academic demands and evolving workforce requirements. This study explores the digital competence of dental students in 2024, with a focus on their self-perceived preparedness for digitally mediated academic and professional environments.

**Methods:**

A cross-sectional study was conducted among 169 dental students at Jundishapur University of Ahvaz using a self-administered questionnaire grounded in the DigComp 2.2 framework. Data were analyzed using descriptive and inferential statistical methods.

**Results:**

Participants reported a relatively high level of self-perceived digital competence (M = 71.55, SD = 12.48) across the eight DigComp 2.2 domains. No statistically significant differences were observed in overall digital competence scores according to gender, age, academic year, or duration of daily internet use, suggesting that these demographic factors were not associated with variations in perceived digital competence within this cohort.

**Conclusions:**

This study provides an exploratory overview of dental students’ self-perceived digital competence based on the DigComp 2.2 framework. The findings highlight domain-specific strengths and weaknesses as perceived by students. Given the self-reported and cross-sectional nature of the data, the results should be interpreted with caution and primarily serve as a basis for future research and curriculum-oriented investigations.

**Supplementary Information:**

The online version contains supplementary material available at 10.1186/s12909-026-08916-2.

## Background

Digital competence has become a fundamental prerequisite for effective participation in contemporary higher education and professional practice, particularly in health-related disciplines where digital technologies increasingly shape clinical decision-making, communication, and lifelong learning. In dental education, students are required to work with digital imaging systems, electronic health records, computer-aided design and manufacturing (CAD/CAM), online scientific resources, and emerging models of teledentistry. Insufficient digital competence may therefore undermine not only academic performance but also graduates’ adaptability to digitally driven clinical environments [1].

Digital competence extends beyond technical skills and encompasses the knowledge, skills, and attitudes necessary to use digital technologies effectively, critically, and responsibly. According to the European Commission, it represents a core lifelong learning competency integrating information literacy, communication and collaboration, content creation, problem-solving, and ethical awareness in digital contexts [2, 3]. Within educational settings, these competencies have been linked to improved learning quality, learner autonomy, and professional preparedness [4].

The COVID-19 pandemic further highlighted the importance of digital competence in dental education, as rapid transitions to online and blended learning revealed considerable variability in students’ readiness to engage with digital learning environments [5, 6]. Although digital competence among university students has been widely studied, much of the literature focuses on Western contexts or on educators rather than learners. Moreover, evidence suggests that students often overestimate their digital abilities, particularly in higher-order domains such as critical evaluation of information and ethical reasoning, underscoring the need for cautious interpretation of self-assessed competence [7].

Research focusing specifically on dental students indicates that discipline-specific digital requirements—including evidence-based decision-making and responsible use of digital clinical tools—pose challenges that are not fully addressed by general digital literacy training [8, 9]. To conceptualize and assess digital competence in a structured manner, several frameworks have been proposed, among which the Digital Competence Framework for Citizens (DigComp) has achieved broad international recognition. The updated DigComp 2.2 framework conceptualizes digital competence across multiple interrelated domains, including information and data literacy, communication and collaboration, digital content creation, safety and ethics, problem-solving, technical operations, and critical thinking [10]. Compared with alternative models, DigComp 2.2 offers greater conceptual clarity and explicit attention to critical and ethical dimensions, making it particularly suitable for health professions education [11].

Previous studies have employed diverse methods—such as questionnaires, performance-based assessments, and observational approaches—to evaluate digital competence among students [12–25]. While participation in digital skills or information literacy training has been associated with higher competence levels [26–28], persistent gaps remain in areas such as advanced information searching, evaluation of online sources, and understanding of legal and ethical issues [29, 30]. Research in dental education remains limited, particularly in non-Western contexts, and few studies have explicitly aligned assessments with the DigComp 2.2 framework.

Against this background, exploratory evidence is needed to understand how dental students perceive their own digital competence within a theoretically grounded framework. Although self-perceived competence does not reflect actual performance, it can provide valuable insights into students’ awareness, confidence, and perceived strengths and weaknesses. Accordingly, the present study aims to explore dental students’ self-perceived digital competence across key DigComp 2.2 domains within an Iranian academic context, addressing an underrepresented disciplinary and regional gap in the existing literature.

### Purpose of the study

The present study aims to evaluate students’ self-assessed digital competence using the DigComp 2.2 framework. Although self-perception cannot substitute for objective performance measures, it provides valuable insights into students’ awareness, confidence, and perceived readiness—particularly in contexts where formal training or performance-based assessment of digital skills is limited. Several previous studies [7, 16, 25] have employed self-report instruments to identify discrepancies between perceived and actual abilities, often serving as an initial step toward subsequent performance-based evaluations or curriculum revisions.

Importantly, this study does not seek to assess students’ objective digital competence. Rather, it focuses on examining students’ self-perceived preparedness and awareness across key digital literacy domains, functioning as a diagnostic and exploratory precursor to curriculum development. Given that digital literacy domains are not yet formally integrated into the dental curriculum at Jundishapur University of Ahvaz, this analysis provides a foundational perspective that may inform future performance-based assessments once structured educational modules are implemented.

Although prior research has emphasized the growing importance of digital competence and information literacy in higher education globally [26–28], relatively few studies have focused specifically on Iranian medical and dental students. Regional evidence suggests that students may overestimate their digital abilities and demonstrate notable gaps in ethical use, information evaluation, and critical thinking [16, 25]. While one Iranian study assessed digital skills among general university students and reported moderate competence levels, it did not address medical or dental contexts nor employ a comprehensive framework such as DigComp 2.2 [31]. Considering the increasing integration of digital tools in dental education and clinical practice, a clear gap remains in the structured evaluation of digital competence among dental students in Iran.

This study addresses that gap by applying the DigComp 2.2 framework, which is widely recognized and aligned with both academic and professional digital skill requirements. The aim is not to provide a performance-based assessment but to understand how dental students perceive their own preparedness for digitally integrated academic and clinical environments. In the absence of a formal digital literacy curriculum at Jundishapur University of Ahvaz, this study represents an initial step toward identifying educational gaps and domains that may benefit from future curriculum development. Accordingly, the research questions are as follows:

1. What is the self-perceived level of digital competence among dental students?
2. Are there statistically significant differences in self-perceived digital competence based on gender, age, academic year, or duration of internet use?

## Methods

### Study design

This exploratory, institution-based, cross-sectional study was designed to provide an initial assessment of dental students’ self-perceived digital competence rather than objectively measured digital skills. Given the exploratory design, no a priori sample size calculation was conducted. The final sample size was determined by the number of eligible students available during the data collection period and their voluntary participation.

The study was conducted among dental students at Jundishapur University of Ahvaz, Iran, during the 2024–2025 academic year. The target population included all third-, fourth-, fifth-, and sixth-year dental students (*N* = 300). Students in earlier academic years were excluded to ensure that participants had sufficient exposure to academic and clinical digital technologies. This exclusion, however, may limit the generalizability of the findings to students in the initial stages of dental education. Accordingly, the study was not designed to detect small subgroup differences.

An invitation to participate was distributed to all eligible students, and responses were received on a voluntary basis. Participation was open to all eligible students, and responses were received across academic years as follows: third year (*n* = 39), fourth year (*n* = 29), fifth year (*n* = 70), and sixth year (*n* = 31). Data were collected between November 15 and December 15, 2024, using a self-administered electronic questionnaire distributed via Google Forms. Participation was voluntary and anonymous.

A total of 300 dental students were invited to participate in the study, of whom 169 completed the questionnaire, yielding a response rate of 56.3%. Although this response rate is comparable to similar survey-based studies in academic settings, the possibility of non-response bias cannot be entirely excluded. As participation was voluntary, it is possible that students with greater interest or confidence in digital technologies were more likely to respond.

All ethical principles related to informed consent, confidentiality, and data protection were strictly observed. Access to the dataset was restricted to the research team. Given the cross-sectional and exploratory design, the findings should be interpreted as indicative rather than confirmatory, particularly with respect to subgroup comparisons.

### Survey instrument

The survey instrument was newly developed for the present study and was conceptually grounded in the Digital Competence Framework for Citizens (DigComp 2.2), which provides a comprehensive structure for describing digital competence across educational contexts [10]. The framework served as a guiding reference to ensure coverage of key digital competence domains relevant to dental education, including information and data literacy, communication and collaboration, digital content use and creation, technical problem-solving, digital citizenship and safety, critical thinking, and lifelong learning. Importantly, the instrument was designed to assess students’ self-perceived digital competence rather than objectively measured or performance-based skills.

The original questionnaire was developed in Persian by the research team based on DigComp 2.2 competence descriptors and adapted to the academic and clinical learning context of dental students. The questionnaire was subsequently translated into English for publication purposes. As the study was exploratory, the translation process did not include formal forward–backward translation or cognitive interviewing. Prior to data collection, the Persian version was reviewed by subject-matter experts in medical education and health information sciences to evaluate face validity, content relevance, and clarity of wording. Minor linguistic refinements were made based on expert feedback.

The final instrument consisted of 17 items grouped into eight DigComp 2.2–aligned dimensions:

1. basic digital skills,
2. information literacy,
3. communication skills,
4. educational content use and creation,
5. technical skills,
6. digital citizenship,
7. critical thinking, and
8. lifelong learning [10].

Given the applied educational context, the instrument included a combination of Likert-scale and dichotomous (Yes/No) items. Likert-type items captured gradations of perceived competence, whereas dichotomous items were used for basic or experience-based skills where a binary response was considered appropriate. Although this mixed-format approach introduces heterogeneity in scaling, it has been used in previous self-assessment studies of digital competence in higher education [32].

Likert-scale items were rated on a four-point scale ranging from 1 (“low”) to 4 (“very high”), with no neutral midpoint to encourage directional judgment. Dichotomous items were coded as 1 (“Yes”) and 0 (“No”). For analytical purposes, item scores within each domain were standardized and averaged to produce domain-level mean scores, thereby allowing comparability across domains despite differences in item format. Higher scores indicated higher levels of self-perceived digital competence.

Internal consistency reliability was assessed using Cronbach’s alpha, yielding an overall coefficient of α = 0.83. Cronbach’s alpha coefficients were as follows: basic skills (α = 0.78), information literacy (α = 0.80), communication skills (α = 0.81), Content creation skills (α = 0.83), technical skills (α = 0.89), digital citizenship (α = 0.82), critical thinking (α = 0.88), and lifelong learning (α = 0.83). However, no assessment of test–retest reliability, construct validity (e.g., factor analysis), or measurement invariance across subgroups was conducted. Consequently, the instrument should be regarded as a preliminary exploratory measure, and further psychometric validation is warranted. Therefore, findings related to domain-level comparisons should be interpreted as indicative patterns rather than definitive psychometric distinctions.

For descriptive purposes only, mean scores were categorized into low, moderate, and high levels of self-perceived digital competence using distribution-based cut-off points, as applied in previous self-assessment studies [32, 33]. These categories do not represent validated competence standards and should not be interpreted as objective indicators of actual digital proficiency. Accordingly, all references to competence levels in this study explicitly refer to perceived competence. The full English version of the questionnaire is provided as a supplementary file.

### Data analysis

Data were analyzed using IBM SPSS Statistics (version 26). Descriptive statistics, including means, standard deviations, frequencies, and percentages, were used to summarize participants’ demographic characteristics and self-perceived digital competence scores across domains.

A post-hoc power analysis conducted using G*Power (version 3.1) indicated that the sample size (*n* = 169) provided approximately 80% power to detect medium effect sizes (Cohen’s d = 0.5; f = 0.25) at α = 0.05. Although the post-hoc analysis suggested adequate power to detect medium effect sizes, the study may still have been underpowered to identify small subgroup differences. Therefore, null findings should be interpreted cautiously.

The normality of domain-level scores was evaluated using visual inspection of histograms and Q–Q plots in conjunction with the Kolmogorov–Smirnov test. As domain-level scores demonstrated approximately normal distributions, parametric statistical tests were applied. Independent-samples t-tests were used for comparisons between binary groups (e.g., gender), while one-way analysis of variance (ANOVA) was employed for comparisons across multiple groups, including academic year and duration of daily internet use.

Prior to conducting independent-samples t-tests and one-way ANOVA, the assumption of homogeneity of variances was evaluated using Levene’s test. In cases where the assumption was violated, appropriate corrections (e.g., Welch’s ANOVA) were considered.

Effect size measures were calculated to facilitate interpretation beyond statistical significance. Cohen’s d was reported for t-tests, and eta-squared (η²) was calculated for ANOVA models. Effect sizes were interpreted using conventional benchmarks to indicate the magnitude and potential practical relevance of observed differences.

Given the exploratory nature of the study and the use of a newly developed self-assessment instrument, no formal adjustment for multiple comparisons (e.g., Bonferroni correction) was applied. Findings were therefore interpreted cautiously, with greater emphasis placed on effect sizes and the consistency of observed patterns rather than on isolated statistically significant results. The absence of statistically significant differences may reflect limited statistical power, restricted intergroup variability, or limited sensitivity of the self-assessment instrument, rather than true homogeneity of digital competence across groups.

All statistical tests were two-tailed, with a significance level set at *p* < 0.05. As completion of all questionnaire items was mandatory in the electronic form, no missing data were observed.

## Results

Of the 300 eligible students invited to participate, 169 completed the questionnaire, yielding a response rate of 56.3%. The mean age of participants was 24.54 years (SD = 3.06). Students from the third to sixth academic years were represented, with the largest proportion enrolled in the fifth year. Detailed demographic characteristics are presented in Table 1.

**Table 1:** Demographics of the participants (*N* = 169)

Variables		*n*	%
Gender	Male	88	52.1
	Female	81	47.9
Age (in years)	23 and under	73	43.2
	24–25	54	32
	More than 25	42	24.9
Academic year	third	39	23.1
	fourth	29	17.2
	fifth	70	41.4
	Sixth and above	31	18.3
Duration of internet use	Up to 3 h	32	18.9
	Up to 6 h	64	37.9
	Up to 9 h	39	23.1
	Up to 12 h	34	20.1

### Digital literacy competence level of dental students

**Fig. 1:**
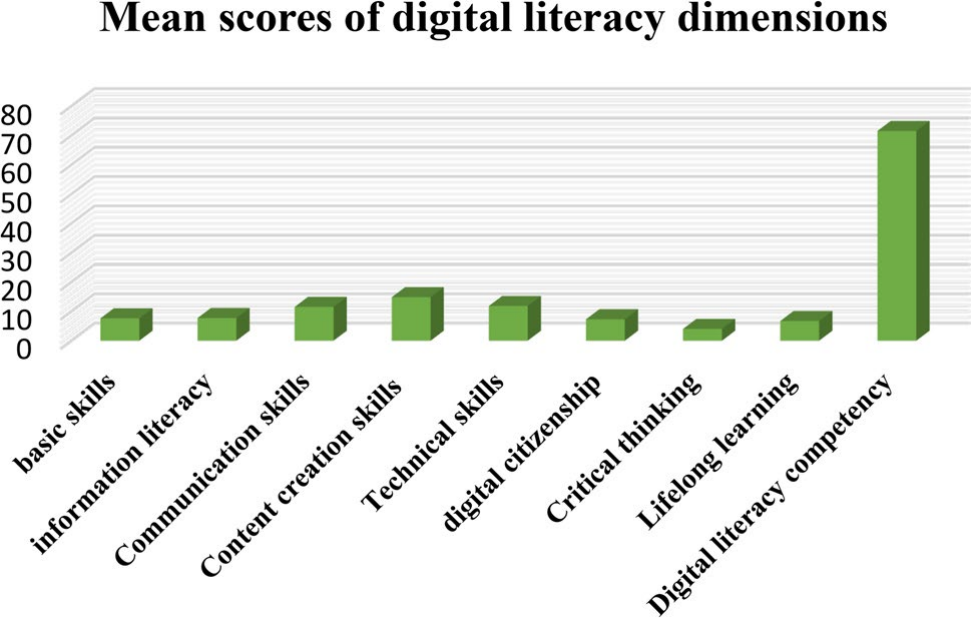
Mean scores of self-perceived digital literacy across competence domains among dental students

Descriptive analysis revealed variability in self-perceived digital literacy across DigComp 2.2–aligned competence domains (Table 2; Fig. 1). Although raw domain scores are presented in Table 2 for descriptive clarity, all inferential statistical analyses were conducted using standardized scores to ensure comparability across domains with differing item counts. The distribution of domain-level scores suggests a heterogeneous pattern of perceived digital competence, with certain applied skill domains demonstrating relatively higher self-assessments compared with more cognitively demanding domains such as critical thinking and information literacy.

**Table 2:** Raw scores of the digital literacy dimensions among dental students based on DigComp 2.2 (*N* = 169)

Dimensions of digital literacy Competency Assessment Instrument	M	SD	Minimum	Maximum
basic skills	7.61	1.96	2	10
information literacy	7.71	1.51	2	10
Communication skills	11.52	2.35	5	15
Content creation skills	14.83	3.39	5	20
Technical skills	11.83	3.55	4	20
digital citizenship	7.29	1.62	3	10
Critical thinking	4.01	0.93	1	5
Lifelong learning	6.70	1.70	2	10
Digital literacy competency	71.55	12.48	30	100

For descriptive purposes, the aggregated self-perceived digital literacy score was located toward the upper end of the distribution. However, this overall pattern coexisted with domain-specific weaknesses, underscoring heterogeneity in perceived digital competence rather than a uniformly high competence profile.

### Differences in digital literacy competency by gender

No statistically significant difference in overall self-perceived digital literacy was observed between male and female students (t(167) = 0.35, *p* = 0.73). The effect size was negligible (Cohen’s d = 0.05), indicating no meaningful practical association between gender and perceived competence (Table 3).

**Table 3:** Gender-based differences in digital literacy competence (*N* = 169)

Gender	*n*	M	SD	t	*p*	Cohen’s d	95% Confidence Interval of the Difference	
male	88	71.88	13.42	0.35	0.73	0.05	Lower	Upper
female	81	71.20	11.42				-3.13	4.50

Cohen’s *d* values of 0.2, 0.5, and 0.8 indicate small, medium, and large effects, respectively

### Differences in digital literacy competency by age

Differences across age groups were not statistically significant (F(2, 166) = 0.23, *p* = 0.79), with a trivial effect size (η² = 0.003), suggesting that age accounted for a negligible proportion of variance (Table 4).

**Table 4:** Age-based differences in digital literacy competency (*N* = 169)

Age	M	SD	F	P	η² (Eta squared)	95% Confidence Interval of the Difference	
						Lower	Upper
23 and under	72.27	11.57	0.23	0.79	0.003	69.57	74.97
						67.68	74.79
24–25	71.24	13.02				66.44	74.97
More than 25	70.70	13.51					

*η*²values of 0.01, 0.06, and 0.14 indicate small, medium, and large effects, respectively

### Differences in digital literacy competency by duration of internet use

Similarly, no statistically significant differences were identified across categories of daily internet use duration (F(3, 165) = 2.20, *p* = 0.09, η² = 0.038). The observed effect size was small (η² = 0.038), indicating limited practical relevance despite minor descriptive variation (Table 5).

**Table 5:** Duration of internet use–based differences in digital literacy competency (*N* = 169)

Duration of internet use	M	SD	F	P	η² (Eta squared)	95% Confidence Interval of the Difference	
						Lower	Upper
Up to 3 h	69.37	13.68	2.20	0.09	0.038	64.44	74.30
Up to 6 h	74.23	13.15				70.92	77.55
Up to 9 h	68.41	10.51				65	71.82
Up to 12 h	72.26	11.37				68.29	76.23

### Differences in digital literacy competency by academic year

**Fig. 2:**
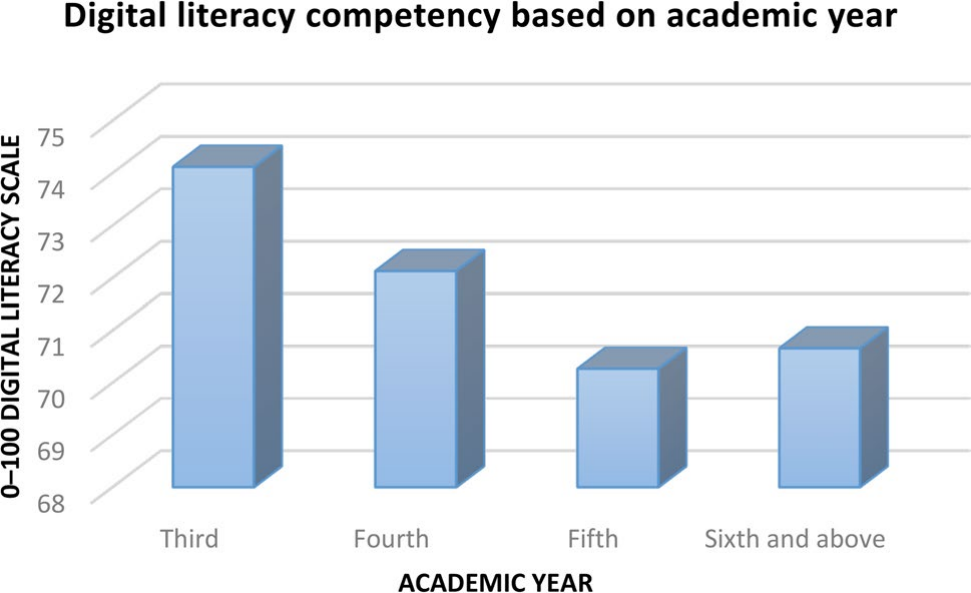
Self-assessed digital literacy competency by academic year

Although third-year students demonstrated descriptively higher mean scores, the overall group comparison did not reach statistical significance (F(3, 165) = 0.86, *p* = 0.45, η² = 0.015). Given the exploratory cross-sectional design and reliance on self-assessment, these differences should not be interpreted as evidence of longitudinal change (Table 6; Fig. 2).

**Table 6:** Academic year–based differences in digital literacy competency (*N* = 169)

Academic year	M	SD	F	P	η² (Eta squared)	95% Confidence Interval of the Difference	
						Lower	Upper
Third	74.12	11.71	0.86	0.45	0.015	70.33	77.92
Fourth	72.13	9.68				68.45	75.82
Fifth	70.27	13.66				67.01	73.53
Sixth and above	70.66	12.95				65.82	75.50

The descriptively higher self-perceived digital literacy scores observed among earlier-year students do not provide evidence of a true decline in digital competence over time. Given the cross-sectional design and reliance on self-assessment, this pattern may reflect differences in self-evaluation, confidence, or exposure to increasingly complex academic and clinical digital tasks rather than actual changes in competence levels.

## Discussions

This exploratory study examined dental students’ self-perceived digital competence using a DigComp 2.2–informed framework and identified a heterogeneous competence profile rather than a uniformly high level of perceived digital readiness. While students reported comparatively higher confidence in domains related to educational content use and creation, technical skills, and communication, consistently lower levels of self-perceived competence were observed in information literacy, digital citizenship, lifelong learning, and most notably critical thinking. Similar uneven patterns of perceived digital competence across domains have been reported among university students in diverse educational contexts, indicating that digital competence is multidimensional rather than a single unified construct [12, 25, 34, 35].

Interpreted within the DigComp 2.2 framework, the observed pattern suggests that dental students may feel more confident in operational and task-oriented digital activities, while perceiving themselves as less competent in higher-order cognitive and evaluative domains. DigComp 2.2 explicitly emphasizes that digital competence extends beyond technical proficiency to encompass critical evaluation of information, ethical engagement, and reflective lifelong learning [10]. Lower scores in these domains therefore point to potential gaps in students’ perceived ability to critically appraise digital information and engage responsibly with digital environments, rather than deficiencies in basic technology use. Comparable domain-level discrepancies—where functional digital skills score higher than evaluative, ethical, and reflective competencies—have been reported among dental, medical, nursing, and pre-service teacher populations [36–39].

Conversely, other studies have reported overall lower levels of perceived digital competence or substantial variability across domains, particularly in information literacy and critical evaluation skills, highlighting the influence of curricular design, institutional culture, and assessment approaches [16, 40–42]. The present findings therefore contribute to a growing body of literature demonstrating that perceived digital competence is context-dependent, unevenly distributed across domains, and sensitive to how competence is conceptualized and measured [43, 44]. Nevertheless, these interpretations remain tentative, as the present study was not designed to test causal mechanisms or objectively verify domain-specific competence.

One notable pattern observed in this study was the coexistence of relatively high aggregated self-perceived digital competence with low scores in critical thinking. This apparent paradox has been widely discussed in educational research and is often interpreted through the concept of the illusion of competence, including the Dunning–Kruger effect, whereby individuals may overestimate their abilities due to limited metacognitive awareness [45]. In digital learning contexts, frequent exposure to technology and routine digital tasks may foster confidence without necessarily translating into deeper evaluative skills or critical understanding [34, 46].

Previous research in dental and health professions education has similarly documented discrepancies between students’ confidence in using digital tools and their ability to critically assess digital information or apply digital skills in complex academic and clinical settings [1, 9, 11, 47]. The present findings align with this literature and reinforce concerns that self-assessment instruments may primarily capture perceived familiarity and routine use rather than higher-order competencies such as critical thinking, ethical reasoning, and responsible digital citizenship [48, 49].

No statistically significant differences in self-perceived digital competence were observed across gender, age, academic year, or duration of daily internet use, and effect sizes were consistently small. Rather than indicating true homogeneity of digital competence, this pattern likely reflects a combination of methodological and contextual factors, including limited statistical power, restricted variability within a single institutional setting, and the limited sensitivity of self-report measures to detect subtle intergroup differences [32, 50, 51]. Similar findings of minimal or inconsistent subgroup differences have been reported in studies of university students’ self-perceived digital competence across disciplines and countries [24, 52].

The descriptively higher self-perceived competence observed among earlier-year students should not be interpreted as evidence of a decline in digital competence over time. Given the cross-sectional design and reliance on self-assessment, this pattern may instead reflect shifts in self-evaluation as students’ progress through increasingly complex academic and clinical environments. Greater exposure to professional responsibilities, ethical challenges, and authentic performance demands may lead senior students to assess their own competence more critically, resulting in lower self-reported scores despite stable or improving actual skills. Similar interpretations have been proposed in prior studies of dental students’ digital and information literacy [8, 53, 54].

The findings therefore reflect perceived digital competence within a specific institutional and cultural context and should not be generalized to dental students nationally or internationally without further multi-center validation. Consequently, the observed domain-level differences may partly reflect measurement characteristics of the instrument rather than purely substantive differences in competence. These considerations underscore the need to interpret the findings within the methodological constraints outlined below.

### Limitations

Several limitations should be considered when interpreting these findings. First, the response rate of 56.3% raises the possibility of non-response bias, as students with greater interest in or confidence about digital technologies may have been more likely to participate. Consequently, the results may overrepresent students with higher perceived competence.

Second, the exclusive reliance on self-reported data introduces susceptibility to social desirability bias, self-perception bias, and potential overestimation of competence. The study assessed perceived digital competence rather than objectively measured performance, and therefore does not provide direct evidence of actual digital proficiency or readiness for digitally mediated clinical practice.

Third, although post-hoc power analysis indicated sufficient power to detect medium effect sizes, the study may have been underpowered to identify small subgroup differences. Accordingly, the absence of statistically significant differences across demographic variables should be interpreted cautiously.

Fourth, the cross-sectional and single-institution design limits internal validity and restricts the generalizability of the findings to other dental education contexts. The results reflect perceived digital competence within a specific institutional and cultural setting and should not be generalized without multi-center replication.

Finally, the newly developed questionnaire underwent limited psychometric evaluation, restricted to internal consistency reliability. The absence of construct validation (e.g., factor analysis), test–retest reliability assessment, and measurement invariance testing constrains the strength of inferences drawn from domain-level comparisons. In addition, the mixed-format structure of the instrument (combining Likert-scale and dichotomous items) may have influenced score distribution and comparability across domains. Therefore, the categorization of competence levels should be regarded as descriptive and sample-dependent rather than as normative indicators of digital proficiency.

### Implications for education and future research

In light of these limitations, the implications for dental education should be interpreted as exploratory rather than prescriptive. Although lower self-perceived competence in critical thinking and digital citizenship may serve as a starting point for hypothesis-driven investigations in future studies, the present findings do not provide sufficient evidence to support specific curricular reforms. Instead, they underscore the need for future research employing validated instruments, objective or performance-based assessments, and longitudinal designs aligned with the DigComp 2.2 framework. Such approaches are necessary to better understand how both perceived and actual digital competence develop across different stages of dental education. Overall, these findings should be regarded as hypothesis-generating rather than confirmatory.

## Conclusions

This exploratory study provides a context-specific assessment of dental students’ self-perceived digital competence within the DigComp 2.2 framework. The findings suggest a heterogeneous competence profile, characterized by comparatively higher confidence in technical and functional digital skills and lower confidence in higher-order domains such as critical thinking, digital citizenship, and lifelong learning.

Within the DigComp 2.2 conceptualization of digital competence, these results highlight the potential value of strengthening higher-order cognitive and ethical domains alongside technical proficiency in dental curricula. However, the absence of statistically significant subgroup differences, together with the coexistence of relatively high aggregated scores and domain-specific weaknesses, underscores the methodological constraints inherent in studies that rely exclusively on self-assessment instruments.

Given the cross-sectional design, single-institution sampling, reliance on self-reported data, and the preliminary psychometric evaluation of the instrument without objective performance measures, no conclusions can be drawn regarding students’ actual readiness for digitally driven dental practice.

## Supplementary Information

Supplementary Material 1.

Supplementary Material 2.

## Data Availability

The datasets generated and/or analyzed during the current study are not publicly available due to confidentiality restrictions but are available from the corresponding author upon reasonable request. The questionnaire developed for this study is included as supplementary file.

## Abbreviations

DigComp: Digital Competence Framework for Citizens
