# Supplementary material for: Digital literacy competence among dental students: an assessment using a DigComp 2.2-based instrument
Source: BMC Med Educ. 2026 May 25;26:1173. doi: 10.1186/s12909-026-08916-2 (PMC13378175; doi:10.1186/s12909-026-08916-2)
Supplement: Supplementary file 1 — Supplementary Material 1. [file 12909_2026_8916_MOESM1_ESM.pdf]

Digital Literacy Competence Questionnaire (Developed for This Study Based on the DigComp 2.2 Framework)

"This questionnaire is designed to assess your self-perceived level of digital competence based on the DigComp 2.2 framework. For each statement, please indicate the extent to which the description reflects your current level of skill or experience. Responses are provided using a four-point Likert scale, defined as follows:

1 = Low – I have minimal familiarity or ability in this area.

2 = Moderate – I have a basic or developing level of ability.

3 = High – I am generally competent and can perform well in most situations.

4 = Very High – I have strong confidence and advanced ability in this area.

Please answer all items as accurately and honestly as possible based on your current experience. There are no right or wrong answers; the purpose is to understand your perceived competence."

Part A. Demographic Information

1. Age: ____
2. Gender: □ Male □ Female □ Prefer not to say
3. Academic year:
   □ Third year □ Fourth year □ Fifth year □ Sixth year
4. Have you previously taken any formal digital skills/computer-related course?
   □ Yes □ No

Part B. Digital Competence Dimensions (Total = 17 items)

Each item is scored either *Yes/No* or on a *4-point Likert scale* depending on the type of skill assessed.

Dimension 1 – Basic Digital Skills

1. I can use operating systems (Windows/Mac) to manage files, folders, and system settings.
   □ Yes □ No

2. I am familiar with commonly used digital systems and platforms required in university coursework (e.g., LMS, educational portals).
   □ Yes □ No

Dimension 2 – Information Literacy

3. My ability to use search engines (e.g., Google, Bing) to search for online academic or scientific information is:

□ 1 □ 2 □ 3 □ 4

4. My ability to search for and retrieve reliable scientific information from scholarly databases (e.g., PubMed, Web of Science, Scopus) is:

□ 1 □ 2 □ 3 □ 4

5. My ability to perform advanced searches—such as using multiple search engines or combining search strategies to locate relevant academic information—is:

□ 1 □ 2 □ 3 □ 4

Dimension 3 – Communication Skills

6. My ability to communicate with faculty or peers using email and academic communication platforms is:
   □ 1 □ 2 □ 3 □ 4

7. My ability to use digital communication tools (e.g., messaging platforms, university portals) for academic purposes is:
   □ 1 □ 2 □ 3 □ 4

Dimension 4 – Educational Content Use and Creation

8. My ability to create digital educational content (e.g., PowerPoint, Canva, infographics) for academic presentations is:
□ 1 □ 2 □ 3 □ 4

9. My familiarity with software used for creating or editing educational materials (e.g., video editing, digital posters) is:
□ 1 □ 2 □ 3 □ 4

Dimension 5 – Technical Skills

10. I can troubleshoot common software errors (e.g., installation issues, login failures, program crashes).
□ Yes □ No

11. I can install required software or updates needed for academic or clinical courses.
□ Yes □ No

Dimension 6 – Digital Citizenship

12. My knowledge of ethical and legal rules related to using and sharing digital information is:
□ 1 □ 2 □ 3 □ 4

13. My understanding of digital privacy principles (e.g., confidentiality of data, secure handling of personal information) is:
□ 1 □ 2 □ 3 □ 4

Dimension 7 – Critical Thinking

14. My ability to verify the accuracy and reliability of scientific information found online is:
□ 1 □ 2 □ 3 □ 4

15. My ability to distinguish between valid scientific content and misleading/fake information on digital platforms is:

□ 1 □ 2 □ 3 □ 4

Dimension 8 – Lifelong Learning

16. I actively use online learning resources (e.g., webinars, MOOCs, instructional videos) to improve my academic or clinical skills.

□ Yes □ No

17. I am interested in learning and adopting new digital tools or technologies related to dentistry.

□ Yes □ No

“This questionnaire was originally developed in Persian and then translated into English for publication purposes.”
