# Supplementary material for: Digital literacy competence among dental students: an assessment using a DigComp 2.2-based instrument
Source: BMC Med Educ. 2026 May 25;26:1173. doi: 10.1186/s12909-026-08916-2 (PMC13378175; doi:10.1186/s12909-026-08916-2)
Supplement: Supplementary file 2 — Supplementary Material 2. [file 12909_2026_8916_MOESM2_ESM.docx]

**Digital literacy competence among dental students: An assessment using a DigComp 2.2-based instrument**

**Abstract**

**Background In the digital age, university students are expected to possess adequate technological competencies to successfully navigate academic demands and evolving workforce requirements. This study explores the digital competence of dental students in 2024, with a focus on their self-perceived preparedness for digitally mediated academic and professional environments.**

**Methods A cross-sectional study was conducted among 169 dental students at Jundishapur University of Ahvaz using a self-administered questionnaire grounded in the DigComp 2.2 framework. Data were analyzed using descriptive and inferential statistical methods.**

**Results Participants reported a relatively high level of self-perceived digital competence (M = 71.55, SD = 12.48) across the eight DigComp 2.2 domains. No statistically significant differences were observed in overall digital competence scores according to gender, age, academic year, or duration of daily internet use, suggesting that these demographic factors were not associated with variations in perceived digital competence within this cohort.**

**Conclusions This study provides an exploratory overview of dental students’ self-perceived digital competence based on the DigComp 2.2 framework. The findings highlight domain-specific strengths and weaknesses as perceived by students. Given the self-reported and cross-sectional nature of the data, the results should be interpreted with caution and primarily serve as a basis for future research and curriculum-oriented investigations.**

**Keywords Information Literacy; Computer Literacy; Dental Students; Self-Assessment; Educational Measurement**

**Background**

Digital competence has become a fundamental prerequisite for effective participation in contemporary higher education and professional practice, particularly in health-related disciplines where digital technologies increasingly shape clinical decision-making, communication, and lifelong learning. In dental education, students are required to work with digital imaging systems, electronic health records, computer-aided design and manufacturing (CAD/CAM), online scientific resources, and emerging models of teledentistry. Insufficient digital competence may therefore undermine not only academic performance but also graduates’ adaptability to digitally driven clinical environments (1).

Digital competence extends beyond technical skills and encompasses the knowledge, skills, and attitudes necessary to use digital technologies effectively, critically, and responsibly. According to the European Commission, it represents a core lifelong learning competency integrating information literacy, communication and collaboration, content creation, problem-solving, and ethical awareness in digital contexts (2, 3). Within educational settings, these competencies have been linked to improved learning quality, learner autonomy, and professional preparedness (4).

The COVID-19 pandemic further highlighted the importance of digital competence in dental education, as rapid transitions to online and blended learning revealed considerable variability in students’ readiness to engage with digital learning environments (5, 6). Although digital competence among university students has been widely studied, much of the literature focuses on Western contexts or on educators rather than learners. Moreover, evidence suggests that students often overestimate their digital abilities, particularly in higher-order domains such as critical evaluation of information and ethical reasoning, underscoring the need for cautious interpretation of self-assessed competence (7).

Research focusing specifically on dental students indicates that discipline-specific digital requirements—including evidence-based decision-making and responsible use of digital clinical tools—pose challenges that are not fully addressed by general digital literacy training (8, 9). To conceptualize and assess digital competence in a structured manner, several frameworks have been proposed, among which the Digital Competence Framework for Citizens (DigComp) has achieved broad international recognition. The updated DigComp 2.2 framework conceptualizes digital competence across multiple interrelated domains, including information and data literacy, communication and collaboration, digital content creation, safety and ethics, problem-solving, technical operations, and critical thinking (10). Compared with alternative models, DigComp 2.2 offers greater conceptual clarity and explicit attention to critical and ethical dimensions, making it particularly suitable for health professions education (11).

Previous studies have employed diverse methods—such as questionnaires, performance-based assessments, and observational approaches—to evaluate digital competence among students (12-25). While participation in digital skills or information literacy training has been associated with higher competence levels (26-28), persistent gaps remain in areas such as advanced information searching, evaluation of online sources, and understanding of legal and ethical issues (29, 30). Research in dental education remains limited, particularly in non-Western contexts, and few studies have explicitly aligned assessments with the DigComp 2.2 framework.

Against this background, exploratory evidence is needed to understand how dental students perceive their own digital competence within a theoretically grounded framework. Although self-perceived competence does not reflect actual performance, it can provide valuable insights into students’ awareness, confidence, and perceived strengths and weaknesses. Accordingly, the present study aims to explore dental students’ self-perceived digital competence across key DigComp 2.2 domains within an Iranian academic context, addressing an underrepresented disciplinary and regional gap in the existing literature.

**Purpose of the study**

The present study aims to evaluate students’ self-assessed digital competence using the DigComp 2.2 framework. Although self-perception cannot substitute for objective performance measures, it provides valuable insights into students’ awareness, confidence, and perceived readiness—particularly in contexts where formal training or performance-based assessment of digital skills is limited. Several previous studies (7, 16, 25) have employed self-report instruments to identify discrepancies between perceived and actual abilities, often serving as an initial step toward subsequent performance-based evaluations or curriculum revisions.

Importantly, this study does not seek to assess students’ objective digital competence. Rather, it focuses on examining students’ self-perceived preparedness and awareness across key digital literacy domains, functioning as a diagnostic and exploratory precursor to curriculum development. Given that digital literacy domains are not yet formally integrated into the dental curriculum at Jundishapur University of Ahvaz, this analysis provides a foundational perspective that may inform future performance-based assessments once structured educational modules are implemented.

Although prior research has emphasized the growing importance of digital competence and information literacy in higher education globally (26-28), relatively few studies have focused specifically on Iranian medical and dental students. Regional evidence suggests that students may overestimate their digital abilities and demonstrate notable gaps in ethical use, information evaluation, and critical thinking (16, 25). While one Iranian study assessed digital skills among general university students and reported moderate competence levels, it did not address medical or dental contexts nor employ a comprehensive framework such as DigComp 2.2 (31). Considering the increasing integration of digital tools in dental education and clinical practice, a clear gap remains in the structured evaluation of digital competence among dental students in Iran.

This study addresses that gap by applying the DigComp 2.2 framework, which is widely recognized and aligned with both academic and professional digital skill requirements. The aim is not to provide a performance-based assessment but to understand how dental students perceive their own preparedness for digitally integrated academic and clinical environments. In the absence of a formal digital literacy curriculum at Jundishapur University of Ahvaz, this study represents an initial step toward identifying educational gaps and domains that may benefit from future curriculum development. Accordingly, the research questions are as follows:

1. What is the self-perceived level of digital competence among dental students?
2. Are there statistically significant differences in self-perceived digital competence based on gender, age, academic year, or duration of internet use?

**Methods**

**Study Design**

This exploratory, institution-based, cross-sectional study was designed to provide an initial assessment of dental students’ self-perceived digital competence rather than objectively measured digital skills. Given the exploratory design, no a priori sample size calculation was conducted. The final sample size was determined by the number of eligible students available during the data collection period and their voluntary participation.

The study was conducted among dental students at Jundishapur University of Ahvaz, Iran, during the 2024–2025 academic year. The target population included all third-, fourth-, fifth-, and sixth-year dental students (N = 300). Students in earlier academic years were excluded to ensure that participants had sufficient exposure to academic and clinical digital technologies. This exclusion, however, may limit the generalizability of the findings to students in the initial stages of dental education. Accordingly, the study was not designed to detect small subgroup differences.

An invitation to participate was distributed to all eligible students, and responses were received on a voluntary basis. Participation was open to all eligible students, and responses were received across academic years as follows: third year (n = 39), fourth year (n = 29), fifth year (n = 70), and sixth year (n = 31). Data were collected between November 15 and December 15, 2024, using a self-administered electronic questionnaire distributed via Google Forms. Participation was voluntary and anonymous.

A total of 300 dental students were invited to participate in the study, of whom 169 completed the questionnaire, yielding a response rate of 56.3%. Although this response rate is comparable to similar survey-based studies in academic settings, the possibility of non-response bias cannot be entirely excluded. As participation was voluntary, it is possible that students with greater interest or confidence in digital technologies were more likely to respond.

All ethical principles related to informed consent, confidentiality, and data protection were strictly observed. Access to the dataset was restricted to the research team. Given the cross-sectional and exploratory design, the findings should be interpreted as indicative rather than confirmatory, particularly with respect to subgroup comparisons.

**Survey instrument**

The survey instrument was newly developed for the present study and was conceptually grounded in the Digital Competence Framework for Citizens (DigComp 2.2), which provides a comprehensive structure for describing digital competence across educational contexts (10). The framework served as a guiding reference to ensure coverage of key digital competence domains relevant to dental education, including information and data literacy, communication and collaboration, digital content use and creation, technical problem-solving, digital citizenship and safety, critical thinking, and lifelong learning. Importantly, the instrument was designed to assess students’ self-perceived digital competence rather than objectively measured or performance-based skills.

The original questionnaire was developed in Persian by the research team based on DigComp 2.2 competence descriptors and adapted to the academic and clinical learning context of dental students. The questionnaire was subsequently translated into English for publication purposes. As the study was exploratory, the translation process did not include formal forward–backward translation or cognitive interviewing. Prior to data collection, the Persian version was reviewed by subject-matter experts in medical education and health information sciences to evaluate face validity, content relevance, and clarity of wording. Minor linguistic refinements were made based on expert feedback.

The final instrument consisted of 17 items grouped into eight DigComp 2.2–aligned dimensions:

(1) basic digital skills,

(2) information literacy,

(3) communication skills,

(4) educational content use and creation,

(5) technical skills,

(6) digital citizenship,

(7) critical thinking, and

(8) lifelong learning (10).

Given the applied educational context, the instrument included a combination of Likert-scale and dichotomous (Yes/No) items. Likert-type items captured gradations of perceived competence, whereas dichotomous items were used for basic or experience-based skills where a binary response was considered appropriate. Although this mixed-format approach introduces heterogeneity in scaling, it has been used in previous self-assessment studies of digital competence in higher education (32).

Likert-scale items were rated on a four-point scale ranging from 1 (“low”) to 4 (“very high”), with no neutral midpoint to encourage directional judgment. Dichotomous items were coded as 1 (“Yes”) and 0 (“No”). For analytical purposes, item scores within each domain were standardized and averaged to produce domain-level mean scores, thereby allowing comparability across domains despite differences in item format. Higher scores indicated higher levels of self-perceived digital competence.

Internal consistency reliability was assessed using Cronbach’s alpha, yielding an overall coefficient of α = 0.83. Cronbach’s alpha coefficients were as follows: basic skills (α = 0.78), information literacy (α = 0.80), communication skills (α = 0.81), Content creation skills (α = 0.83), technical skills (α = 0.89), digital citizenship (α = 0.82), critical thinking (α = 0.88), and lifelong learning (α = 0.83). However, no assessment of test–retest reliability, construct validity (e.g., factor analysis), or measurement invariance across subgroups was conducted. Consequently, the instrument should be regarded as a preliminary exploratory measure, and further psychometric validation is warranted. Therefore, findings related to domain-level comparisons should be interpreted as indicative patterns rather than definitive psychometric distinctions.

For descriptive purposes only, mean scores were categorized into low, moderate, and high levels of self-perceived digital competence using distribution-based cut-off points, as applied in previous self-assessment studies (32, 33). These categories do not represent validated competence standards and should not be interpreted as objective indicators of actual digital proficiency. Accordingly, all references to competence levels in this study explicitly refer to perceived competence. The full English version of the questionnaire is provided as a supplementary file.

**Data analysis**

Data were analyzed using IBM SPSS Statistics (version 26). Descriptive statistics, including means, standard deviations, frequencies, and percentages, were used to summarize participants’ demographic characteristics and self-perceived digital competence scores across domains.

A post-hoc power analysis conducted using G*Power (version 3.1) indicated that the sample size (n = 169) provided approximately 80% power to detect medium effect sizes (Cohen’s d = 0.5; f = 0.25) at α = 0.05. Although the post-hoc analysis suggested adequate power to detect medium effect sizes, the study may still have been underpowered to identify small subgroup differences. Therefore, null findings should be interpreted cautiously.

The normality of domain-level scores was evaluated using visual inspection of histograms and Q–Q plots in conjunction with the Kolmogorov–Smirnov test. As domain-level scores demonstrated approximately normal distributions, parametric statistical tests were applied. Independent-samples t-tests were used for comparisons between binary groups (e.g., gender), while one-way analysis of variance (ANOVA) was employed for comparisons across multiple groups, including academic year and duration of daily internet use.

Prior to conducting independent-samples t-tests and one-way ANOVA, the assumption of homogeneity of variances was evaluated using Levene’s test. In cases where the assumption was violated, appropriate corrections (e.g., Welch’s ANOVA) were considered.

Effect size measures were calculated to facilitate interpretation beyond statistical significance. Cohen’s d was reported for t-tests, and eta-squared (η²) was calculated for ANOVA models. Effect sizes were interpreted using conventional benchmarks to indicate the magnitude and potential practical relevance of observed differences.

Given the exploratory nature of the study and the use of a newly developed self-assessment instrument, no formal adjustment for multiple comparisons (e.g., Bonferroni correction) was applied. Findings were therefore interpreted cautiously, with greater emphasis placed on effect sizes and the consistency of observed patterns rather than on isolated statistically significant results. The absence of statistically significant differences may reflect limited statistical power, restricted intergroup variability, or limited sensitivity of the self-assessment instrument, rather than true homogeneity of digital competence across groups.

All statistical tests were two-tailed, with a significance level set at p < 0.05. As completion of all questionnaire items was mandatory in the electronic form, no missing data were observed.

**Results**

Of the 300 eligible students invited to participate, 169 completed the questionnaire, yielding a response rate of 56.3%. The mean age of participants was 24.54 years (SD = 3.06). Students from the third to sixth academic years were represented, with the largest proportion enrolled in the fifth year. Detailed demographic characteristics are presented in Table 1.

**Table 1** Demographics of the participants (N = 169)

| **Variables** | | **n** | **%** |
| --- | --- | --- | --- |
| Gender | Male | 88 | 52.1 |
|  | Female | 81 | 47.9 |
| Age (in years) | 23 and under | 73 | 43.2 |
|  | 24-25 | 54 | 32 |
|  | More than 25 | 42 | 24.9 |
| Academic year | third | 39 | 23.1 |
|  | fourth | 29 | 17.2 |
|  | fifth | 70 | 41.4 |
|  | Sixth and above | 31 | 18.3 |
| Duration of internet use | Up to 3 hours | 32 | 18.9 |
|  | Up to 6 hours | 64 | 37.9 |
|  | Up to 9 hours | 39 | 23.1 |
|  | Up to 12 hours | 34 | 20.1 |

**Digital literacy competence level of dental students**

Descriptive analysis revealed variability in self-perceived digital literacy across DigComp 2.2–aligned competence domains (Table 2; Fig. 1). Although raw domain scores are presented in Table 2 for descriptive clarity, all inferential statistical analyses were conducted using standardized scores to ensure comparability across domains with differing item counts. The distribution of domain-level scores suggests a heterogeneous pattern of perceived digital competence, with certain applied skill domains demonstrating relatively higher self-assessments compared with more cognitively demanding domains such as critical thinking and information literacy.

**Table 2** Raw scores of the digital literacy dimensions among dental students based on DigComp 2.2 (N = 169)

| **Dimensions of digital literacy Competency Assessment Instrument** | **M** | **SD** | **Minimum** | **Maximum** |
| --- | --- | --- | --- | --- |
| basic skills | 7.61 | 1.96 | 2 | 10 |
| information literacy | 7.71 | 1.51 | 2 | 10 |
| Communication skills | 11.52 | 2.35 | 5 | 15 |
| Content creation skills | 14.83 | 3.39 | 5 | 20 |
| Technical skills | 11.83 | 3.55 | 4 | 20 |
| digital citizenship | 7.29 | 1.62 | 3 | 10 |
| Critical thinking | 4.01 | 0.93 | 1 | 5 |
| Lifelong learning | 6.70 | 1.70 | 2 | 10 |
| Digital literacy competency | 71.55 | 12.48 | 30 | 100 |

Fig. 1 Mean scores of self-perceived digital literacy across competence domains among dental students

For descriptive purposes, the aggregated self-perceived digital literacy score was located toward the upper end of the distribution. However, this overall pattern coexisted with domain-specific weaknesses, underscoring heterogeneity in perceived digital competence rather than a uniformly high competence profile.

**Differences in digital literacy competency by gender**

No statistically significant difference in overall self-perceived digital literacy was observed between male and female students (t(167) = 0.35, p = 0.73). The effect size was negligible (Cohen’s d = 0.05), indicating no meaningful practical association between gender and perceived competence (Table 3).

**Table 3** Gender-based differences in digital literacy competence (N = 169)

| **Gender** | **n** | **M** | **SD** | **t** | **p** | **Cohen’s d^[[1]](#footnote-1)^** | **95% Confidence Interval of the Difference** | |
| --- | --- | --- | --- | --- | --- | --- | --- | --- |
| male | 88 | 71.88 | 13.42 | 0.35 | 0.73 | 0.05 | Lower | Upper |
| female | 81 | 71.20 | 11.42 |  |  |  | -3.13 | 4.50 |

**Differences in digital literacy competency by age**

Differences across age groups were not statistically significant (F(2, 166) = 0.23, p = 0.79), with a trivial effect size (η² = 0.003), suggesting that age accounted for a negligible proportion of variance (Table 4).

**Table 4** Age-based differences in digital literacy competency (N = 169)

| **Age** | **M** | **SD** | **F** | **P** | **η² (Eta squared)^[[2]](#footnote-2)^** | **95% Confidence Interval of the Difference** | |
| --- | --- | --- | --- | --- | --- | --- | --- |
|  |  |  |  |  |  | Lower | Upper |
| 23 and under | 72.27 | 11.57 | 0.23 | 0.79 | 0.003 | 69.57 | 74.97 |
|  |  |  |  |  |  | 67.68 | 74.79 |
| 24-25 | 71.24 | 13.02 |  |  |  | 66.44 | 74.97 |
| More than 25 | 70.70 | 13.51 |  |  |  |  |  |

**Differences in digital literacy competency by** **duration of internet use**

Similarly, no statistically significant differences were identified across categories of daily internet use duration (F(3, 165) = 2.20, p = 0.09, η² = 0.038). The observed effect size was small (η² = 0.038), indicating limited practical relevance despite minor descriptive variation (Table 5).

**Table 5** Duration of internet use–based differences in digital literacy competency (N = 169)

| **Duration of internet use** | **M** | **SD** | **F** | **P** | **η² (Eta squared)** | **95% Confidence Interval of the Difference** | |
| --- | --- | --- | --- | --- | --- | --- | --- |
|  |  |  |  |  |  | Lower | Upper |
| Up to 3 hours | 69.37 | 13.68 | 2.20 | 0.09 | 0.038 | 64.44 | 74.30 |
| Up to 6 hours | 74.23 | 13.15 |  |  |  | 70.92 | 77.55 |
| Up to 9 hours | 68.41 | 10.51 |  |  |  | 65 | 71.82 |
| Up to 12 hours | 72.26 | 11.37 |  |  |  | 68.29 | 76.23 |

**Differences in digital literacy competency by academic year**

Although third-year students demonstrated descriptively higher mean scores, the overall group comparison did not reach statistical significance (F(3, 165) = 0.86, p = 0.45, η² = 0.015). Given the exploratory cross-sectional design and reliance on self-assessment, these differences should not be interpreted as evidence of longitudinal change (Table 6; Fig. 2).

**Table 6** Academic year–based differences in digital literacy competency (N = 169)

| **Academic year** | **M** | **SD** | **F** | **P** | **η² (Eta squared)** | **95% Confidence Interval of the Difference** | |
| --- | --- | --- | --- | --- | --- | --- | --- |
|  |  |  |  |  |  | Lower | Upper |
| Third | 74.12 | 11.71 | 0.86 | 0.45 | 0.015 | 70.33 | 77.92 |
| Fourth | 72.13 | 9.68 |  |  |  | 68.45 | 75.82 |
| Fifth | 70.27 | 13.66 |  |  |  | 67.01 | 73.53 |
| Sixth and above | 70.66 | 12.95 |  |  |  | 65.82 | 75.50 |

**Fig. 2** Self-assessed digital literacy competency by academic year

The descriptively higher self-perceived digital literacy scores observed among earlier-year students do not provide evidence of a true decline in digital competence over time. Given the cross-sectional design and reliance on self-assessment, this pattern may reflect differences in self-evaluation, confidence, or exposure to increasingly complex academic and clinical digital tasks rather than actual changes in competence levels.

**Discussions**

This exploratory study examined dental students’ self-perceived digital competence using a DigComp 2.2–informed framework and identified a heterogeneous competence profile rather than a uniformly high level of perceived digital readiness. While students reported comparatively higher confidence in domains related to educational content use and creation, technical skills, and communication, consistently lower levels of self-perceived competence were observed in information literacy, digital citizenship, lifelong learning, and most notably critical thinking. Similar uneven patterns of perceived digital competence across domains have been reported among university students in diverse educational contexts, indicating that digital competence is multidimensional rather than a single unified construct (12, 25, 34, 35).

Interpreted within the DigComp 2.2 framework, the observed pattern suggests that dental students may feel more confident in operational and task-oriented digital activities, while perceiving themselves as less competent in higher-order cognitive and evaluative domains. DigComp 2.2 explicitly emphasizes that digital competence extends beyond technical proficiency to encompass critical evaluation of information, ethical engagement, and reflective lifelong learning (10). Lower scores in these domains therefore point to potential gaps in students’ perceived ability to critically appraise digital information and engage responsibly with digital environments, rather than deficiencies in basic technology use. Comparable domain-level discrepancies—where functional digital skills score higher than evaluative, ethical, and reflective competencies—have been reported among dental, medical, nursing, and pre-service teacher populations (36-39).

Conversely, other studies have reported overall lower levels of perceived digital competence or substantial variability across domains, particularly in information literacy and critical evaluation skills, highlighting the influence of curricular design, institutional culture, and assessment approaches (16, 40-42). The present findings therefore contribute to a growing body of literature demonstrating that perceived digital competence is context-dependent, unevenly distributed across domains, and sensitive to how competence is conceptualized and measured (43, 44). Nevertheless, these interpretations remain tentative, as the present study was not designed to test causal mechanisms or objectively verify domain-specific competence.

One notable pattern observed in this study was the coexistence of relatively high aggregated self-perceived digital competence with low scores in critical thinking. This apparent paradox has been widely discussed in educational research and is often interpreted through the concept of the illusion of competence, including the Dunning–Kruger effect, whereby individuals may overestimate their abilities due to limited metacognitive awareness (45). In digital learning contexts, frequent exposure to technology and routine digital tasks may foster confidence without necessarily translating into deeper evaluative skills or critical understanding (34, 46).

Previous research in dental and health professions education has similarly documented discrepancies between students’ confidence in using digital tools and their ability to critically assess digital information or apply digital skills in complex academic and clinical settings (1, 9, 11, 47). The present findings align with this literature and reinforce concerns that self-assessment instruments may primarily capture perceived familiarity and routine use rather than higher-order competencies such as critical thinking, ethical reasoning, and responsible digital citizenship (48, 49).

No statistically significant differences in self-perceived digital competence were observed across gender, age, academic year, or duration of daily internet use, and effect sizes were consistently small. Rather than indicating true homogeneity of digital competence, this pattern likely reflects a combination of methodological and contextual factors, including limited statistical power, restricted variability within a single institutional setting, and the limited sensitivity of self-report measures to detect subtle intergroup differences (32, 50, 51). Similar findings of minimal or inconsistent subgroup differences have been reported in studies of university students’ self-perceived digital competence across disciplines and countries (24, 52).

The descriptively higher self-perceived competence observed among earlier-year students should not be interpreted as evidence of a decline in digital competence over time. Given the cross-sectional design and reliance on self-assessment, this pattern may instead reflect shifts in self-evaluation as students’ progress through increasingly complex academic and clinical environments. Greater exposure to professional responsibilities, ethical challenges, and authentic performance demands may lead senior students to assess their own competence more critically, resulting in lower self-reported scores despite stable or improving actual skills. Similar interpretations have been proposed in prior studies of dental students’ digital and information literacy (8, 53, 54).

The findings therefore reflect perceived digital competence within a specific institutional and cultural context and should not be generalized to dental students nationally or internationally without further multi-center validation. Consequently, the observed domain-level differences may partly reflect measurement characteristics of the instrument rather than purely substantive differences in competence. These considerations underscore the need to interpret the findings within the methodological constraints outlined below.

**Limitations**

Several limitations should be considered when interpreting these findings. First, the response rate of 56.3% raises the possibility of non-response bias, as students with greater interest in or confidence about digital technologies may have been more likely to participate. Consequently, the results may overrepresent students with higher perceived competence.

Second, the exclusive reliance on self-reported data introduces susceptibility to social desirability bias, self-perception bias, and potential overestimation of competence. The study assessed perceived digital competence rather than objectively measured performance, and therefore does not provide direct evidence of actual digital proficiency or readiness for digitally mediated clinical practice.

Third, although post-hoc power analysis indicated sufficient power to detect medium effect sizes, the study may have been underpowered to identify small subgroup differences. Accordingly, the absence of statistically significant differences across demographic variables should be interpreted cautiously.

Fourth, the cross-sectional and single-institution design limits internal validity and restricts the generalizability of the findings to other dental education contexts. The results reflect perceived digital competence within a specific institutional and cultural setting and should not be generalized without multi-center replication.

Finally, the newly developed questionnaire underwent limited psychometric evaluation, restricted to internal consistency reliability. The absence of construct validation (e.g., factor analysis), test–retest reliability assessment, and measurement invariance testing constrains the strength of inferences drawn from domain-level comparisons. In addition, the mixed-format structure of the instrument (combining Likert-scale and dichotomous items) may have influenced score distribution and comparability across domains. Therefore, the categorization of competence levels should be regarded as descriptive and sample-dependent rather than as normative indicators of digital proficiency.

**Implications for education and future research**

In light of these limitations, the implications for dental education should be interpreted as exploratory rather than prescriptive. Although lower self-perceived competence in critical thinking and digital citizenship may serve as a starting point for hypothesis-driven investigations in future studies, the present findings do not provide sufficient evidence to support specific curricular reforms. Instead, they underscore the need for future research employing validated instruments, objective or performance-based assessments, and longitudinal designs aligned with the DigComp 2.2 framework. Such approaches are necessary to better understand how both perceived and actual digital competence develop across different stages of dental education. Overall, these findings should be regarded as hypothesis-generating rather than confirmatory.

**Conclusions**

This exploratory study provides a context-specific assessment of dental students’ self-perceived digital competence within the DigComp 2.2 framework. The findings suggest a heterogeneous competence profile, characterized by comparatively higher confidence in technical and functional digital skills and lower confidence in higher-order domains such as critical thinking, digital citizenship, and lifelong learning.

Within the DigComp 2.2 conceptualization of digital competence, these results highlight the potential value of strengthening higher-order cognitive and ethical domains alongside technical proficiency in dental curricula. However, the absence of statistically significant subgroup differences, together with the coexistence of relatively high aggregated scores and domain-specific weaknesses, underscores the methodological constraints inherent in studies that rely exclusively on self-assessment instruments.

Given the cross-sectional design, single-institution sampling, reliance on self-reported data, and the preliminary psychometric evaluation of the instrument without objective performance measures, no conclusions can be drawn regarding students’ actual readiness for digitally driven dental practice.

**List of Abbreviations**

DigComp Digital Competence Framework for Citizens

**Declarations**

**Trial registration:**

**Not applicable.**

**Ethics approval and consent to participate**

Ethics approval was granted by the Ethics Committee of Ahvaz Jundishapur University of Medical Sciences (IR.AJUMS.REC.1402.307). All participating students provided informed consent. This study was conducted in accordance with the Declaration of Helsinki and all relevant institutional guidelines and regulations.

**Consent for publication**

Not applicable.

**Availability of data and materials**

The datasets generated and/or analyzed during the current study are not publicly available due to confidentiality restrictions but are available from the corresponding author upon reasonable request. The questionnaire developed for this study is included as supplementary file.

**Competing interests**

The authors declare that they have no competing interests.

**Funding**

No funding was received to conduct this study.

**Authors’ contributions**

MSh conceived the study idea and proposed the research topic.

FK analyzed and interpreted the data, wrote the main text of the manuscript, and served as the corresponding author.

SMM reviewed the manuscript critically for important intellectual content and approved the final version.

MA collected data from dental students and contributed to data organization.

All authors read and approved the final manuscript.

**Acknowledgements**

Not applicable.

**References**

1. Saxena P, Gupta SK, Mehrotra D, Kamthan S, Sabir H, Katiyar P, et al. Assessment of digital literacy and use of smart phones among Central Indian dental students. J Oral Biol Craniofac Res. 2018;8(1):40-3.

DOI:10.1016/j.jobcr.2017.10.001

2. Carretero S, Vuorikari R, Punie Y. DigComp 2.1. The digital competence framework for citizens with eight proficiency levels and examples of use2017.

DOI:10.2760/38842

3. European Commission. KEY COMPETENCES FOR LIFELONG LEARNING. 2019.

4. Levano-Francia L, Sanchez, S., Guillén-Aparicio, P., , Tello-Cabello S, Herrera-Paico, N., Collantes-Inga, Z. . Digital Competences and Education2019; 7(2). Available from: <https://files.eric.ed.gov/fulltext/EJ1220548.pdf>

doi.org/10.20511/pyr2019.v7n2.329

(Accessed May 20, 2024).

5. Chang TY, Hsu ML, Kwon JS, Kusdhany MLS, Hong G. Effect of online learning for dental education in asia during the pandemic of COVID-19. J Dent Sci. 2021;16(4):1095-101.

DOI:10.1016/j.jds.2021.06.006

6. Whitelaw S, Mamas MA, Topol E, Van Spall HGC. Applications of digital technology in COVID-19 pandemic planning and response. Lancet Digit Health. 2020;2(8):e435-e40.

DOI:10.1016/s2589-7500(20)30142-4

7. Sciumbata F. Students of humanities and digital skills: a survey on Italian university students. 2020;7-27.

DOI:10.6092/issn.2532-8816/9877

8. Sedky RAF, Ben Dor B, Mustafa DS, Galal MM, Nour KA, Anwar MN, et al. Self-assessment skills of undergraduate students in operative dentistry: Preclinical performance and gender. Dent Med Probl. 2025;62(6):1035-42.

DOI:10.17219/dmp/175276

9. Tok Ö N, Yurttaş AN, Taş S. YouTube videos as an information source about exercises for temporomandibular disorders. Dent Med Probl. 2024;61(6):855-63.

DOI:10.17219/dmp/170922

10. Vuorikari R, Kluzer, S. and Punie, Y. DigComp 2.2: The Digital Competence Framework for Citizens - With new examples of knowledge, skills and attitudes. Luxembourg: Publications Office of the European Union, Luxembourg; 2022.

DOI:10.2760/115376, JRC128415.

11. Waligóra J, Ryniewicz J, Bereziewicz W, Bereziewicz J, Gajdzica M, Pytko-Polończyk J. Quality of education and the evaluation of its outcomes in tooth preparation among dental students in Poland. Dent Med Probl. 2025;62(4):671-9.

DOI:10.17219/dmp/192749

12. Alonso-García S, Victoria Maldonado J, Martínez-Domingo J-A, Berral-Ortiz B. Analysis of self-perceived digital competences in future educators: A study at the university of Granada. Journal of Technology and Science Education. 2024;144.

DOI:10.3926/jotse.2521

13. Borysova S, Zadorina O, Kotiash I, Bukoros A. Digital Competencies in Ukrainian Education of the Future: Teaching and Assessment. Futurity Education. 2023;3(4):217-31.

DOI:10.57125/FED.2023.12.25.13

14. Cabezas-González M, Martín S. Social Educators: A Study of Digital Competence from a Gender Differences Perspective / Socijalni pedagozi: istraživanje digitalne kompetencije iz perspektive spolnih razlika. Croatian Journal of Education - Hrvatski časopis za odgoj i obrazovanje. 2018;20.

DOI:10.15516/cje.v20i1.2632

15. Fernández Sánchez M, Silva J. Evaluación de la competencia digital de futuros docentes desde una perspectiva de género. RIED-Revista Iberoamericana de Educación a Distancia. 2022;25.

DOI:10.5944/ried.25.2.32128

16. Irfan N, Rafiq M, Arif M. Information competency assessment of undergraduates: A Pakistani perspective. IFLA Journal. 2024;50.

DOI:10.1177/03400352231222040

17. Li Z, Slavkova O, Gao Y. Role of digitalization, digital competence, and parental support on performance of sports education in low-income college students. Front Psychol. 2022;13979318.

DOI:10.3389/fpsyg.2022.979318

18. Luján Guevara GJ, Apolaya Sotelo JP. Currículo por competencias en educación universitaria: retos y perspectivas de la educación virtual en el contexto de la Emergencia Sanitaria Nacional. SCIÉNDO. 2021;24(1):27-33.

DOI:10.17268/sciendo.2021.003

19. Mehrvarz M, Heidari E, Farrokhnia M, Noroozi O. The mediating role of digital informal learning in the relationship between students' digital competence and their academic performance. Computers & Education. 2021;167104184.

DOI:<https://doi.org/10.1016/j.compedu.2021.104184>

20. Mendez Gijon F. Diseño de un ambiente de aprendizaje blended learning como propuesta de innovación educativa en la Universidad de la Sierra Juárez. RIDE Revista Iberoamericana para la Investigación y el Desarrollo Educativo. 2020;11.

DOI:10.23913/ride.v11i21.731

21. Morales-Zambrano FF, Pazmiño-Campuzano MF, San Andrés-Laz EM. Competencias digitales de los docentes en la educación media del Ecuador. Polo del conocimiento. 2021;6(2):18.

DOI:<https://doi.org/10.23857/pc.v6i2.2246>

22. R G, Sadagopan D. INFORMATION LITERACY ASSESSMENT AMONG STUDENTS – MADURAI KAMARAJ UNIVERSITY AND MANONMANIAM SUNDARANAR UNIVERSITY. 2018.

23. Tomaš S, Vrdoljak M, Kalebić Jakupčević K. Digital Skills Assessment and Digital Competences Self-Assessment Among Students at the University of Split. Journal of Elementary Education. 2024;17(1):53-68.

DOI:10.18690/rei.3084

24. Wild S, Schulze Heuling L. How do the Digital Competences of Students in Vocational Schools Differ from Those of Students in Cooperative Higher Education Institutions in Germany? Empirical Research in Vocational Education and Training. 2020.

DOI:10.1186/s40461-020-00091-y

25. Zhao Y, Sánchez-Gómez MC, Pinto-Llorente A, Zhao L. Digital Competence in Higher Education: Students’ Perception and Personal Factors. Sustainability. 2021;1312184.

DOI:10.3390/su132112184

26. Boh Podgornik B, Dolničar D, Sorgo A, Bartol T. Evaluation of Information Literacy of Slovenian University Students2015.

DOI:10.1007/978-3-319-28197-1_50C

27. Nakaziba S, Kaddu S, Namuguzi M, Mwanzu A. Exploring experiences regarding information literacy competencies among nursing students at Aga Khan University, Uganda. Library Management. 2022;44.

DOI:10.1108/LM-08-2022-0071

28. Özden G, Çevi̇k Aktura S, Gul E, Çıtlık Sarıtaş S. The information literacy levels of nursing students who take the course on information techniques. 2020;27846.

DOI:10.5455/annalsmedres.2019.10.654

29. Kozhevnikova O. Distant learning and digital competence of Russian students2013.

30. Rafique G, Khan H. Information Literacy Skills of Management Sciences' Students. Pakistan Journal of Information Management and Libraries. 2017;19.

DOI:10.47657/2017191014

31. Hashemi S, Faqihian Alirezaei Alawiche R. Investigating the Relationship between Motivational Beliefs and Digital Literacy among Bushehr University Students. Iranian Journal of Information Management. 2023;9(1):204-16.

DOI:10.22034/aimj.2023.218281

32. Hatlevik O, Christophersen K-A. Digital competence at the beginning of upper secondary school: Identifying factors explaining digital inclusion. Computers & Education. 2013;63240–7.

DOI:10.1016/j.compedu.2012.11.015

33. Rodríguez De Dios I, van Oosten J, Igartua JJ. A Study of the Relationship between Parental Mediation and Adolescents’ Digital Skills, Online Risks and Online Opportunities. Computers in Human Behavior. 2018;82.

DOI:10.1016/j.chb.2018.01.012

34. Morgan A, Sibson R, Jackson D. Digital demand and digital deficit: conceptualising digital literacy and gauging proficiency among higher education students. Journal of Higher Education Policy and Management. 2022;441-18.

DOI:10.1080/1360080X.2022.2030275

35. Peng D, Yu Z. A Literature Review of Digital Literacy over Two Decades. Education Research International. 2022;20221-8.

DOI:10.1155/2022/2533413

36. Çebi A, Reisoğlu İ. Digital Competence: A Study from the Perspective of Pre-service Teachers in Turkey. Journal of New Approaches in Educational Research. 2020;9294.

DOI:10.7821/naer.2020.7.583

37. Martzoukou K, Luders E, Mair J, Kostagiolas P, Johnson N, Work F, et al. A cross‐sectional study of discipline‐based self‐perceived digital literacy competencies of nursing students. Journal of Advanced Nursing. 2023;80n/a-n/a.

DOI:10.1111/jan.15801

38. Erdilek D, Gümüştaş B, Güray Efes B. Digitalization era of dental education: A systematic review. Dent Med Probl. 2023;60(3):513-25.

DOI:10.17219/dmp/156804

39. Razdan P, Das A, Habiba S, Doley S, Tiwari DA, Hazari P. Knowledge, perception and attitude of dentists regarding the role of artificial intelligence in the field of pediatric dentistry: An online questionnaire study. Dent Med Probl. 2025;62(4):645-55.

DOI:10.17219/dmp/183901

40. Sunny SK, Ramasamy K. Digital literacy skills of students of Sacred Heart College, Chalakudy: an empirical study. Journal of Applied Research in Higher Education. 2024;ahead-of-print(ahead-of-print).

DOI:10.1108/JARHE-06-2023-0257

41. Nguyen LAT, Habók A. Digital Literacy of EFL Students: An Empirical Study in Vietnamese Universities. Libri. 2021;7253 - 66.

DOI:DOI:10.1515/libri-2020-0165

42. Ukwoma SC, Iwundu N, Iwundu I. Digital literacy skills possessed by students of UNN, implications for effective learning and performance: A study of the MTN Universities Connect Library. New Library World. 2016;117702-20.

DOI:10.1108/NLW-08-2016-0061

43. Siddiq F, Gochyyev P, Wilson M. Learning in Digital Networks – ICT literacy: A novel assessment of students' 21st century skills. Computers & Education. 2017;10911-37.

DOI:<https://doi.org/10.1016/j.compedu.2017.01.014>

44. Tzafilkou K, Perifanou M, Economides A. Development and validation of students’ digital competence scale (SDiCoS). International Journal of Educational Technology in Higher Education. 2022;19.

DOI:10.1186/s41239-022-00330-0

45. Kruger J, & Dunning, D. . Unskilled and unaware of it: How difficulties in recognizing one's own incompetence lead to inflated self-assessments. Journal of Personality and Social Psychology. 1999;77(6):13.

DOI: <https://doi.org/10.1037/0022-3514.77.6.1121>

46. Tran T, Ho M-T, Pham T-H, Nguyen M-H, Nguyen K-LP, Vuong T-T, et al. How Digital Natives Learn and Thrive in the Digital Age: Evidence from an Emerging Economy. Sustainability. 2020;12(9):3819.

DOI:doi:10.3390/su12093819

47. Frings D, Sykes S, Ojo A, Rowlands G, Trasolini A, Dadaczynski K, et al. Differences in digital health literacy and future anxiety between health care and other university students in England during the COVID-19 pandemic. BMC Public Health. 2022;22(1):658.

DOI:10.1186/s12889-022-13087-y

48. Abdulai AF, Tiffere AH, Adam F, Kabanunye MM. COVID-19 information-related digital literacy among online health consumers in a low-income country. Int J Med Inform. 2021;145104322.

DOI:10.1016/j.ijmedinf.2020.104322

49. Kaarakainen M-T, Kivinen O, Vainio T. Performance-based testing for ICT skills assessing: a case study of students and teachers’ ICT skills in Finnish schools. Universal Access in the Information Society. 2018;17.

DOI:<https://doi.org/10.1007/s10209-017-0553-9>

50. Aesaert K, van Braak J. Gender and socioeconomic related differences in performance based ICT competences. Computers & Education. 2015;848-25.

DOI:<https://doi.org/10.1016/j.compedu.2014.12.017>

51. Cabezas-González M, Martín S, Sanches-Ferreira M, Diogo F. Do gender and age affect the level of digital competence? A study with University students. Fonseca, Journal of Communication. 2017;15115-32.

DOI:10.14201/fjc201715115132

52. Taib A, Awang, Yunita, Mohamed, Shazalina Shuhidan, Zakaria, Zaiza Norsuriati Zainal, Sulistyowati, Sri, Ifada, Luluk Muhimatul. Digitalization of the accounting profession: An assessment of digital competencies in a Malaysian comprehensive university. Asian Journal of University Education (AJUE) 2023;19(2):16.

DOI:10.14738/assrj.115.16814

53. Kousar M, Mahmood K, editors. Information Literacy Skills Assessment of Undergraduate Engineering Students. Worldwide Commonalities and Challenges in Information Literacy Research and Practice; 2013 2013//; Cham: Springer International Publishing.

DOI:[10.1007/978-3-319-03919-0_63](https://doi.org/10.1007/978-3-319-03919-0_63?urlappend=%3Futm_source%3Dresearchgate.net%26utm_medium%3Darticle)

54. Guevara G, Sotelo J. Curriculum by competences in university education: challenges and perspectives of virtual education in the context of the National Health Emergency. SCIÉNDO. 2021;2427-33.

DOI:10.17268/sciendo.2021.003

1. . Cohen’s d values of 0.2, 0.5, and 0.8 indicate small, medium, and large effects, respectively. [↑](#footnote-ref-1)
2. . *η² values of 0.01, 0.06, and 0.14 indicate small, medium, and large effects, respectively.* [↑](#footnote-ref-2)
